# Editorial: Enhancing agricultural water management: techniques for improving crop water efficiency and sustainability

**DOI:** 10.3389/fpls.2025.1655207

**Published:** 2025-08-07

**Authors:** Qi Wu, Yidi Sun, Hanmi Zhou

**Affiliations:** ^1^ College of Water Conservancy, Shenyang Agricultural University, Shenyang, Liaoning, China; ^2^ College of Hydraulic Science and Engineering, Yangzhou University, Yangzhou, Jiangsu, China; ^3^ College of Agricultural Equipment Engineering, Henan University of Science and Technology, Luoyang, China

**Keywords:** precision irrigation, emission mitigation, drought resilience, nitrogen-water interactions, soil health

The global agricultural landscape faces unprecedented challenges: escalating water scarcity, climate volatility, and the urgent need to reconcile productivity with environmental stewardship. This Research Topic, *“Enhancing Agricultural Water Management: Techniques for Improving Crop Water Efficiency and Sustainability,”* presents cutting-edge research that addresses these dual imperatives. The 13 studies compiled herein—spanning diverse crops, climates, and management systems—collectively advance our understanding of how precision water and nutrient management can optimize yields while minimizing ecological footprints.

## 1 Core themes and innovations

### 1.1 Precision irrigation as a game-changer

Subsurface drip irrigation (SDI) emerges as a linchpin strategy. Studies on alfalfa (Ma et al.) and apples (Chen et al.) demonstrate SDI’s ability to enhance water use efficiency (WUE) by delivering water directly to root zones, reducing evaporation. Notably, SDI depth profoundly influences apple quality—a critical insight for arid horticulture. Similarly, work in oasis cotton fields (Zhang et al.) reveals biodegradable mulch films synergize with optimized irrigation quotas to improve soil hydrothermal conditions over multi-year cycles. The novel finding that daily minimum leaf turgor pressure reliably indicates apple tree water status (Chen et al.) further empowers precision irrigation scheduling.

### 1.2 Nitrogen-water synergies and emission mitigation

The delicate balance between water, nitrogen (N), and greenhouse gas emissions is dissected in alfalfa systems (Ma et al.). Optimized SDI combined with N management slashes N_2_O emissions by 19–32% while maintaining forage yields—a blueprint for low-carbon forage production. Iron-modified biochar (Zhang et al.) offers another breakthrough, adsorbing phosphorus and reducing P fertilizer needs by 20% in peanut fields. Sweet corn as summer catch crop can reduce nitrate leaching in the sweet cherry greenhouses (Hou et al.). These innovations prove that resource efficiency and emission reduction are achievable simultaneously.

### 1.3 Drought resilience and adaptive strategies

Arid-zone agriculture demands crop-specific adaptations. For Northwest China’s alfalfa (Ma et al.), optimized irrigation methods redistribute water and nitrogen toward deeper roots, bolstering drought resilience. Wheat studies (Zhang et al.) establish critical soil moisture thresholds governing carbon assimilate redistribution and grain formation—enabling targeted irrigation during sensitive growth stages. Cotton’s “dry sowing and wet emergence” technique exemplifies how strategic water timing enhances photosynthesis and yield in water-scarce environments (Ding et al.).

### 1.4 Organic amendments and soil health

Organic fertilizers’ role transcends nutrient supply: they rebuild soil structure and enhance water retention. Pumpkin production increased by 7.01%–25.26% with organic fertilization, linked to improved soil organic carbon and microbial activity (Ren et al.). However, pumpkin yield initially increased and then decreased in response to increasing organic fertilizer application (Yin et al.). Maize trials further confirm that aeration coupled with organic inputs alleviate soil compaction in drylands, boosting yields by up to 30% (Yu et al.).

### 1.5 Modeling and threshold-driven management

Predictive tools are vital for scalability. The evaluation of nine canopy resistance models identifies optimal approaches for estimating wheat evapotranspiration using the Penman-Monteith equation (Wu et al.). Mediterranean kiwifruit orchards leverage root-uptake dynamics and soil moisture thresholds to enable precision irrigation scheduling—reducing water use by 25% without yield loss (Calabritto et al.). Meanwhile, a water and solute transport HYDRUS-1D model was used to evaluate the effects of using sweet corn as a catch crop on deep water drainage and nitrate leaching in a sweet cherry greenhouse soil, guiding sustainable irrigation decisions (Hou et al.).

## 2 Cross-cutting implications

### 2.1 Water-smart technologies are location- and crop-specific

SDI excels in perennial systems (alfalfa, orchards), while moisture thresholds and modeling suit annual crops. Biodegradable mulches prove ideal for cotton in oases, whereas organic amendments shine in vegetable systems. Context is paramount.

### 2.2 Synergistic practices maximize co-benefits

The most successful interventions combine multiple levers:

SDI + optimized N management reduces emissions *and* conserves water.

Organic fertilizer coupled with precision irrigation improves soil health *and* crop quality.

Modeling + sensor-based thresholds enable predictive adaptation.

### 2.3 From field to policy

These studies provide actionable intelligence:

Policymakers should incentivize SDI in water-stressed regions and subsidize organic/slow-release fertilizers.

Farmers can adopt moisture thresholds and modeling tools for real-time decisions.

Researchers must expand long-term trials (e.g., 3+ years) to validate sustainability.

### 2.4 Future frontiers

While this Research Topic makes strides, knowledge gaps persist:

1. Economic Viability: Cost-benefit analyses of SDI/organic amendments at scale.

2. Digital Integration: IoT sensors + AI for dynamic irrigation-nutrient management.

3. Salinity Interactions: Water efficiency in salt-affected soils.

4. Global South Applications: Adapting techniques for smallholder systems.

## 3 Concluding remarks

Agriculture cannot thrive by prioritizing yield alone; it must harmonize productivity with planetary boundaries. The work in this Research Topic illuminates a path forward—one where every drop of water and gram of fertilizer is leveraged with precision. By embracing science-backed water management, we transform agriculture from a resource-intensive sector into a beacon of efficiency and resilience. The future of food security hinges on our ability to scale these innovations, and this Research Topic provides the empirical foundation to do so.

